# Add-on radioiodine during long-term BRAF/MEK inhibition in patients with RAI-refractory thyroid cancers: a reasonable option?

**DOI:** 10.1007/s12020-023-03388-6

**Published:** 2023-05-16

**Authors:** Filipe Miguel Montes de Jesus, Vittoria Espeli, Gaetano Paone, Luca Giovanella

**Affiliations:** 1 Clinic for Nuclear Medicine and Molecular Imaging, Imaging Institute of Southern Switzerland, Ente Ospedaliero Cantonale, Bellinzona, Switzerland; 2 Medical Oncology, Oncology Institute of Southern Switzerland, Ente Ospedaliero Cantonale, Mendrisio, Switzerland; 3 Clinic for Nuclear Medicine, University Hospital of Zürich, Zürich, Switzerland

**Keywords:** Differentiated thyroid carcinoma, Dedifferentiation, Radioiodine, Radioiodine-refractory-differentiated thyroid carcinoma

## Abstract

Dual modulation of the MAPK pathway with BRAF (e.g., dabrafenib) and MEK (e.g., trametinib) inhibitors has the potential to re-establish radioiodine (RAI) sensitivity in BRAF-mutated RAI-refractory (RAI-R)-differentiated thyroid carcinoma (DTC) cells. Here we showed that (1) double BRAF/MEK inhibition may still reach a significant redifferentiation in patients with a long-history RAI-R DTC and multiple previous treatments; (2) the addition of high RAI activities may obtain a significant structural response in such patients; and (3) a divergence between increasing thyroglobulin and structural response may be a reliable biomarker or redifferentiation. Accordingly, the add-on prescription of high activities of 131I should be considered in RAI-R patients under multikinase inhibitors with stable or responding structural disease and divergent increase of Tg levels.

## Introduction

**Fig. 1:**
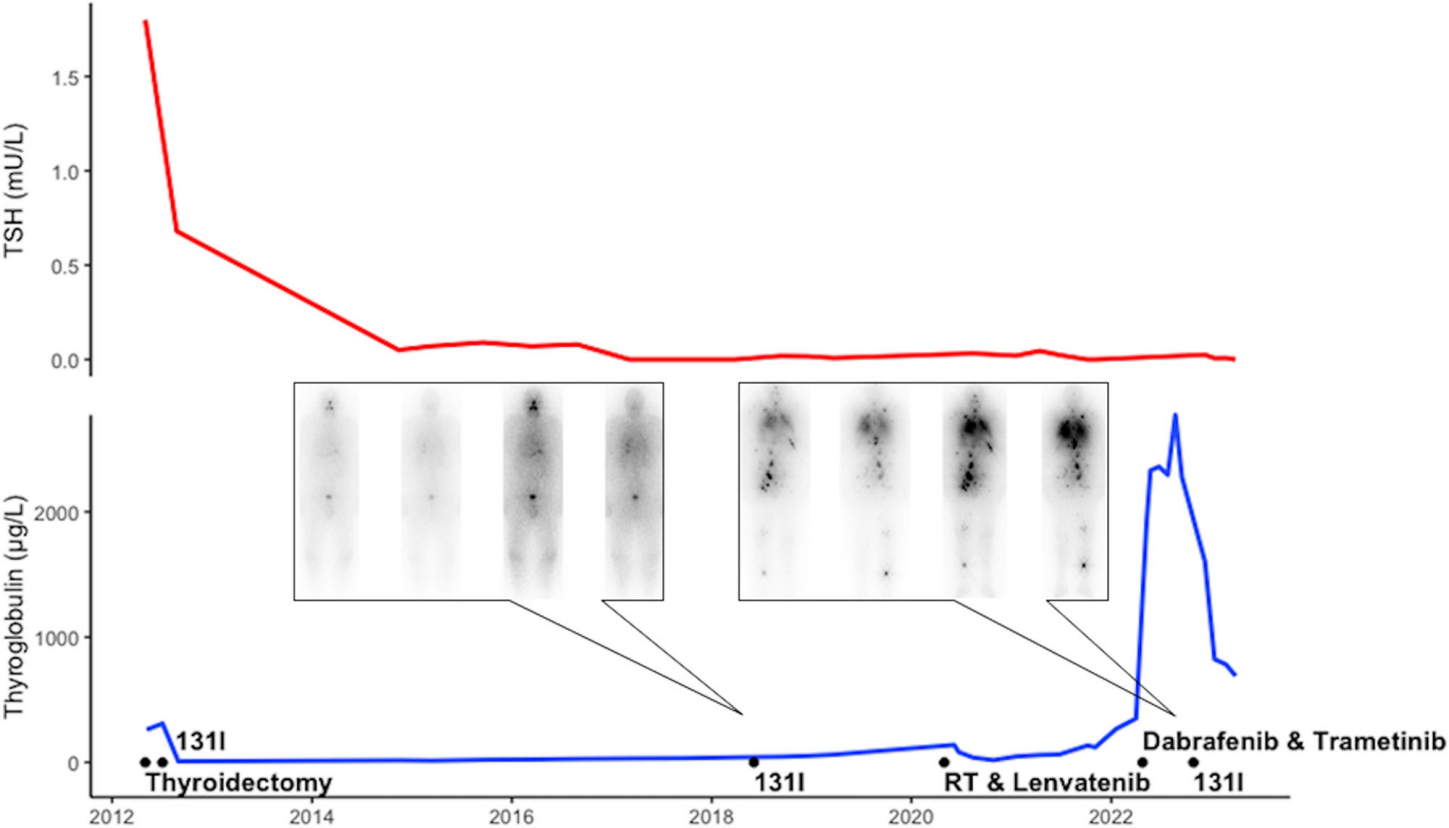
Overview of biochemical and imaging findings

Radioactive active iodine (RAI) therapy is the standard of care for ablation or adjuvant postoperative therapy of differentiated thyroid cancer (DTC). Moreover, administration of high activities of RAI is a mainstay to treat distant metastases from DTC and was the first available and effective targeted therapy for metastatic disease [1]. In addition, the early toxicity is low, especially when compared to that observed with other oncological treatments, and long-term increased risk of secondary leukemia and cancers is only observed in some patients following the administration of high cumulative RAI activities (>22 GBq) [2]. Unfortunately, large retrospective studies have shown that up to 60% of metastatic patients become radioactive iodine-refractory (RAI-R) over time, mainly due to a loss of differentiation of the thyroid cancer cells with impaired radioiodine uptake and intracellular retention [3]. The introduction of multi-kinase inhibitors has improved outcomes in RAI-R patients and brought new clinical insights. Landmark trials SELECT and DECISION, with lenvatinib and sorafenib, respectively, have demonstrated significant improvements in progression free survival and its use is now recommended in major guidelines with an ESMO-Magnitude of Clinical Benefit Scale of A [4–8]. Second-line systemic therapy is further dependent on the genetic profiling of the tumor. Alterations in the MAPK [MAPK kinase (MEK)/ERK] signaling pathway, such as mutant BRAF, RAS, and RET fusions represent the majority of alterations in differentiated thyroid cancers and are key drivers in the progression of disease [9]. Furthermore, BRAF V600E mutations have been implicated in decreased expression of sodium iodide symporters (NIS), contributing to radioactive iodine refractoriness. Dual modulation of the MAPK signaling pathway with BRAF inhibitors (e.g., dabrafenib) and MEK inhibitors (e.g., trametinib) have the potential to restore NIS function and re-establish RAI sensitivity (i.e., redifferentiation) [10]. Initial murine studies demonstrated the efficacy of MAPK pathway inhibitors to increase tumor cells’ susceptibility to RAI [11]. Increased awareness of this phenomenon has led to the first reports and human trials [12]. Yet, due to the rarity of condition and the lack of a standardized approach, data is lacking to help clinicians select the most suitable patients, determine adequate doses and plan timing of the treatment when attempting RAI-R re-differentiation. Recently, we observed a 63-year-old male diagnosed in April 2012 with a FDG-active multifocal papillary thyroid carcinoma, including FDG-active cervical lymph node and FDG-negative small pulmonary metastasis on staging **[**^**18**^F]FDG-PET/CT [cT2(m), cN1b, cM1 (lung)]. Initial work-up revealed a BRAF V600E mutated tumor and a pre-operatory thyroglobulin level of 262 μg/L (normal value < 58 μg/L) (Fig. 1). Patient underwent a total thyroidectomy with central and bi-lateral lymph node resection and surgical pathology examination reported a multifocal PTC with minimal extra-thyroid extension and multiple lymph node metastases in both central and bi-lateral compartments [pT3a(m), pN1b, pMx, cM1 (lung)]. Subsequent treatment with ^131^I [7.4 GBq per os] was performed and post-treatment whole body scintigraphy imaging revealed a small remnant of thyroid tissue without focal uptakes outside the thyroid bed. Compared to pre-operative examination the post-treatment [^18^F]FDG-PET/CT demonstrated the disappearance of thyroid lesions and cervical lymph node metastases (i.e., compete surgical resection of local and loco-regional tumor tissues) and unchanged FDG-negative lung metastases, with a corresponding thyroglobulin level at 8.9 μg/L (i.e., incomplete structural response). Accordingly, a diagnosis of metastatic RAI-R PTC was formulated at that point. An active surveillance strategy was advised due to the excellent performance status and quality of life of the patient and his own preferences. Lung metastases remained morphologically stable (i.e., number and size) and FDG-inactive until 2014, when an increase in number and size of lung metastases was reported, with a simultaneous appearance of FDG uptake in most lesions and a corresponding increase of thyroglobulin level (i.e., 17.21 μg/L). Throughout follow-up thyroglobulin levels as well as number, size and FDG uptake of lung metastases slowly increased without any impact on the quality of life of our patient. Thyroglobulin level reached a concentration of 79 μg/L by mid-2018. At this point a radioiodine whole-body scintigraphy revealed focal areas of low to moderate radioiodine uptake in some lung nodules and we decided to proceed with a second cycle of ^131^I treatment [7.4 GBq]. The corresponding PT-WBS confirmed a mild uptake of ^131^I by lung lesions previously visualized at diagnostic WBS but most lesion remained not radioiodine avid. Despite a decrease of thyroglobulin levels from 79 to 46 μg/L (September 2018) a structural and FDG-metabolic progression of lung metastases associated with new lymph node, soft tissue (muscle) and skeletal lesions was demonstrated by the [^18^F]FDG-PET/CT examination performed in September 2018. The patients remained, however, completely asymptomatic with an excellent performance status and quality of life. Therefore, active surveillance under TSH-suppressive therapy was continued. Unfortunately, a T11 vertebral lesion at risk of fracture was detected in 2020 prompting a stereotactic external beam radiation therapy (total delivered dose 30 Gy). Furthermore, the patient was started on a standard dose of lenvatinib 24 mg/day dose from May 2020. The lenvatinib dose was then lowered to 10 mg/day due to side effects (mainly hypertension, fatigue, loss of appetite with 15 kg weight loss and hand-foot syndrome). The disease remained biochemically and morphologically stable until mid-2021, when the thyroglobulin levels started to increase at each consequent follow-up reaching 350 μg/L in April 2022. The corresponding [^18^F]FDG-PET/CT also revealed an increase in the number and metabolic rate of metastatic lesions. Thereafter, lenvatinib was discontinued and a next generation molecular sequencing of the primary tumor performed. The presence of BRAF V600E mutation was confirmed, without other mutations nor gene fusions, and the patient was started with dabrafenib (150 mg/2xday) and trametinib (2 mg/day) off-label, after approval of the insurance company. Two months after start of therapy, which was well tolerated, a partial metabolic response was achieved on [^18^F]FDG-PET/CT (July 2022), with a dramatic increase in thyroglobulin levels (2330 μg/L). The patient continued with the initial dose of dabrafenib and trametinib for the following months, after extension of the approval by the insurance company. The follow-up [^18^F]FDG-PET/CT in September 2022 showed stable structural disease with an unremarkable faint increase of FDG-avidity of the skeletal and lymph nodal lesions with further increase in the thyroglobulin levels (2776 μg/L). Considering the divergence between imaging data and thyroglobulin trend, redifferentiation of RAI-R disease was hypothesized, and we decided to exploit the redifferentiation potential of BRAF/MEK inhibitors. Afterward, the patient was scheduled to undergo ^131^I therapy with 7.4 GBq. Post-therapy whole-body scintigraphy revealed multiple, intensely radioiodine-avid, lesions at the level of the lymph nodes, lungs and musculoskeletal metastases. Six months after treatment a partial biochemical response was recorded with a thyroglobulin decrease from 2276 to 825 μg/L. Moreover, a partial morphological and metabolic response of the metastatic lesions was observed on the [^18^F]FDG-PET/CT of February 2023.

## Discussion

Current literature on redifferentiation of RAI-refractory thyroid cancers has been recently reviewed by van Nostrand et al. [13]. From their work, only few cases have been identified to use a combination of BRAF and MEK inhibitors [14–16]. Owing to heterogenous methodologies, including different therapeutic targets and redifferentiation procedures, results have varied significantly. Overall, success rate for redifferentiation in larger studies (>8 patients) have varied from 33 and 95% with reported response rates to ^131^I between 33 and 100% [12, 14, 15, 17–19]. Results from a phase II randomized trial comparing dabrafenib monotherapy to dabrafenib + trametinib in patients with BRAF-mutated radioactive iodine refractory DTC have reported an objective response rate of 48% (modified RECIST) in dabrafenib + trametinib arm versus 42% in dabrafenib arm, respectively [20]. This represents a significant improvement compared to the 10% response rate of cabozantinb, which is the recommended drug as second line treatment in DTC without genetic test targeting actionable cancer mutations [8]. These promising results support recommendations from major guidelines to initiate dabrafenib with trametinib treatment in patients with BRAF V600E-mutant disease and open the doors to simplified regimens with dabrafenib alone, respectively [7, 21]. Overall, however, due to the rarity of this condition, optimal selection of patients, timing of treatment and schedules remains challenging. Several studies have suggested that an increase in thyroglobulin levels during targeted therapy may indicate redifferentiation [15, 18]. In our patient, the rapid increase in thyroglobulin after the start of dabrafenib/trametinib with subsequent proven redifferentiation may support this hypothesis and indicate that therapy with radioiodine may be feasible in cases of stable imaging and increasing thyroglobulin in RAI-R patients under targeted therapies. Redifferentiation, however, may not directly translate into treatment efficacy and response rates have varied even with clearly positive whole body scintigraphy scans [13]. Although we had evidence of radioactive iodine uptake, we have only achieved a partial, even if dramatic, metabolic and biochemical response. The response to radioactive iodine therapy is likely influenced by clonal heterogeneity of the tumor determined, in turn, by different signaling pathways and other biological factors. An escalation of administrated activities may further improve the released dose to the tumor and may be achieved by personalized dosimetry. The introduction of ^124^I PET/CT into clinical practice, as earlier studies have highlighted, may allow a personalized administration of higher activities of RAI. Yet, however, ^124^I is not standardly used outside of clinical trials and data supporting the superiority of personalized dosimetry protocols over conventional fixed activities schedules are not available. Accordingly, the highest therapeutic activity authorized in Switzerland (i.e., 7.4 GBq) was administered to our patient achieving a structural response (even if partial) not observed with multi-kinase drugs alone, in line with literature reports. Accordingly, the addition of RAI to multi-kinase inhibitors (i.e., add-on therapy) may augment the effectiveness of the latters without significant additional side effect being RAI well-tolerated especially in comparison with multi-kinase inhibitors. Reliable biomarkers of redifferentiation are warranted to properly identify patients potentially benefitting of these protocols. An increasing thyroglobulin in front of a structural response (either stable disease or structural response) was effective in our case to select our patients making thyroglobulin a promising candidate clinical biomarker of redifferentiation during multi-kinase inhibitors therapy.

## Conclusions

Basing on the reviewed literature and our own clinical experience, we can share some considerations. First, even after many years of RAI-R DTC history and multiple treatments including approved, first line, thyrosin-kinase inhibitors, it is still possible to reach a significant redifferentiation by second line multi-kinase inhibitors and their combination. Second, a divergence between increasing thyroglobulin and structural response may be a reliable biomarker or redifferentiation. Third, the addition of high activities of ^131^I may obtain significant structural responses in such patients. Accordingly, the add-on prescription of high activities of ^131^I should be considered in RAI-R patients under multikinase inhibitors with stable or responding structural disease and divergent increase of Tg levels. Further studies are urgently warranted to clarify the role of serum Tg as a surrogate marker of redifferentiation in such cases. Finally, BRAF/MEK inhibitors may not be available in all centers and all stakeholders, including insurance companies, should be aware of the potential benefits of this therapy in this relatively rare but critical patient population.

## Data Availability

The study was conducted in accordance with applicable regulations. For more information on the study and data sharing, qualified researchers may contact the corresponding author, Prof. Dr. Luca Giovanella, MD PhD (luca.giovanella@eoc.ch).

## References

[1] Ylli D, Van Nostrand D, Wartofsky L (2019). Conventional radioiodine therapy for differentiated thyroid cancer. Endocrinol. Metab. Clin. North Am..

[2] Avram AM, Giovanella L, Greenspan B (2022). SNMMI procedure Standard/EANM practice guideline for nuclear medicine evaluation and therapy of differentiated thyroid cancer: abbreviated version. J. Nucl. Med..

[3] L. Giovanella, D. van Nostrand. Advanced differentiated thyroid cancer: when to stop radioiodine? Q. J. Nucl. Med. Mol. Imaging **63**, 267–270 (2019) 10.23736/S1824-4785.19.03191-1 31271271

[4] Schlumberger M, Tahara M, Wirth LJ (2015). Lenvatinib versus placebo in radioiodine-refractory thyroid cancer. N. Engl. J. Med..

[5] Brose MS, Nutting CM, Jarzab B (2014). Sorafenib in radioactive iodine-refractory, locally advanced or metastatic differentiated thyroid cancer: a randomised, double-blind, phase 3 trial. Lancet.

[6] Fugazzola L, Elisei R, Fuhrer D, Jarzab B, Leboulleux S, Newbold K, Smit J (2019). 2019 European Thyroid Association guidelines for the treatment and follow-up of advanced radioiodine-refractory thyroid cancer. Eur. Thyroid J..

[7] Haddad RI, Bischoff L, Ball D (2022). Thyroid carcinoma, version 2.2022, NCCN clinical practice guidelines in oncology. J. Natl. Compr. Cancer Netw..

[8] Filetti S, Durante C, Hartl DM, Leboulleux S, Locati LD, Newbold K, Papotti MG, Berruti A (2022). ESMO Clinical Practice Guideline update on the use of systemic therapy in advanced thyroid cancer. Ann. Oncol..

[9] Buffet C, Wassermann J, Hecht F, Leenhardt L, Dupuy C, Groussin L, Lussey-Lepoutre C (2020). Redifferentiation of radioiodine-refractory thyroid cancers. Endocr. Relat. Cancer.

[10] Cabanillas ME, Ryder M, Jimenez C (2019). Targeted therapy for advanced thyroid cancer: kinase inhibitors and beyond. Endocr. Rev..

[11] Chakravarty D, Santos E, Ryder M (2011). Small-molecule MAPK inhibitors restore radioiodine incorporation in mouse thyroid cancers with conditional BRAF activation. J. Clin. Invest.

[12] Ho AL, Grewal RK, Leboeuf R (2013). Selumetinib-enhanced radioiodine uptake in advanced thyroid cancer. N. Engl. J. Med..

[13] D. Van Nostrand, I. Vetysman, K. Kulkarni, S.L. Heimlich, K.D. Burman. Redifferentiation of differentiated thyroid cancer: clinical insights from a narrative review of literature. Thyroid 10.1089/thy.2022.0632 (2023) 10.1089/thy.2022.0632 36792922

[14] Leboulleux S, Cao CD, Zerdoud S (2021). MERAIODE: a redifferentiation phase II trial with trametinib and dabrafenib followed by radioactive iodine administration for metastatic radioactive iodine refractory differentiated thyroid cancer patients with a BRAFV600E mutation (NCT 03244956). J. Endocr. Soc..

[15] Jaber T, Waguespack SG, Cabanillas ME (2018). Targeted therapy in advanced thyroid cancer to resensitize tumors to radioactive iodine. J. Clin. Endocrinol. Metab..

[16] Iravani A, Solomon B, Pattison DA, Jackson P, Ravi Kumar A, Kong G, Hofman MS, Akhurst T, Hicks RJ (2019). Mitogen-activated protein kinase pathway inhibition for redifferentiation of radioiodine refractory differentiated thyroid cancer: an evolving protocol. Thyroid.

[17] Rothenberg SM, McFadden DG, Palmer EL, Daniels GH, Wirth LJ (2015). Redifferentiation of iodine-refractory BRAF V600E-mutant metastatic papillary thyroid cancer with dabrafenib. Clin. Cancer Res..

[18] Dunn LA, Sherman EJ, Baxi SS (2019). Vemurafenib redifferentiation of BRAF mutant, RAI-refractory thyroid cancers. J. Clin. Endocrinol. Metab..

[19] Anekpuritanang T, Uataya M, Claimon A, Laokulrath N, Pongsapich W, Pithuksurachai P (2021). The association between radioiodine refractory in papillary thyroid carcinoma, sodium/iodide symporter expression, and BRAFV600E mutation. Onco Targets Ther..

[20] Busaidy NL, Konda B, Wei L (2022). Dabrafenib Versus Dabrafenib + Trametinib in BRAF -Mutated Radioactive Iodine Refractory Differentiated Thyroid Cancer: Results of a Randomized, Phase 2, Open-Label Multicenter Trial. Thyroid.

[21] Filetti S, Durante C, Hartl D, Leboulleux S, Locati LD, Newbold K, Papotti MG, Berruti A (2019). Thyroid cancer: ESMO Clinical Practice Guidelines for diagnosis, treatment and follow-up. Ann. Oncol..

